# Neuropsychological assessment methodology revisited: metatheoretical reflections

**DOI:** 10.3389/fpsyg.2023.1170283

**Published:** 2023-11-17

**Authors:** Josh Joseph Ramminger, Martin Peper, Alexander Nicolai Wendt

**Affiliations:** ^1^Department of Psychology, University of Marburg, Marburg, Germany; ^2^Department of Psychology, Humboldt University of Berlin, Berlin, Germany; ^3^Faculty of Psychology, Sigmund Freud University, Vienna, Austria; ^4^Department of Psychology, Heidelberg University, Heidelberg, Germany

**Keywords:** neuropsychological assessment, lense model paradigm in neuropsychology, philosophy of science, phenomenological psychology, metatheory, ecological validity, modularity, neural networks

## Abstract

Theory building in neuropsychology, similar to other disciplines, rests on metatheoretical assumptions of philosophical origin. Such assumptions regarding the relation of psychological and physiological variables influence research methodologies as well as assessment strategies in fields of application. Here, we revisit the classic procedure of Double Dissociation (DD) to illustrate the connection of metatheory and methodology. In a seemingly unbridgeable opposition, the classical neuropsychological procedure of DD can be understood as either presupposing localizationism and a modular view of the brain, or as a special case of the generalized neuro-lens model for neuropsychological assessment. In the latter case, it is more easily compatible with a perspective that emphasizes the systemic-network, rather than the modular, nature of the brain, which as part of the organism, proportionately mediates the situatedness of the human being in the world. This perspective not only makes it possible to structure ecological validation processes and give them a metatheoretical foundation, but also to interlace it with the phenomenological insight that the laboratory as one context of empirical research may be analyzed in terms of situated experience. We conclude with showing that both the localizationist and the system science approach can agree on a view of the brain as a dynamical network, and that metatheory may thus offer important new perspectives of reconciliation.

## The problem of consciousness and neuropsychological methodology

The contemporary discourse surrounding the issue of low replicability rates in psychology (Open Science Collaboration, 2015) posits that such rates can be attributed, at least in part, to deficiencies in theory building (Muthukrishna and Henrich, 2019; Oberauer and Lewandowsky, 2019; Witte, 2022). Therefore, the validity of empirical research is contingent upon the soundness of scientific theory. Scientific theories encompass convictions pertaining to the subject-matter under investigation, as well as the interrelationships between the various entities or attributes being examined (Borgstede and Eggert, 2022). To the extent that these are metatheoretical or ontological, they also belong to the scope of philosophy (cf. Hastings et al., 2020). However, it should be noted that a metatheoretical framework differs from a specific scientific theory in that it can structure competing concrete individual theories (Muthukrishna and Henrich, 2019). Metatheoretical frameworks that belong to the relation of psychological and physiological variables are coherently also found in psychology, physiology, and cognitive neuroscience (cf. Marom, 2020; Pauen, 2021). These disciplines thus take a stance toward a problem which philosophers call the mind-body problem or problem of consciousness (see Pauen, 2021; Schleim, 2022). Yet, throughout history, philosophers could not achieve a consensus on the *solvability* of this problem. In 1872, Du Bois-Reymond gave a series of lectures on the limits of scientific explanation, one of these limits being the problem of consciousness (Du Bois-Reymond, 1872; see also Schleim, 2022). In 2013 Kügler has regarded the “ever-shifting problem” of consciousness as an unsolvable riddle (Kügler, 2013; but see Pauen, 2021). Regardless of matters concerning the solvability of the problem, recent work in theoretical psychology and neuroscience has emphasized that philosophical positions or metatheoretical frameworks, such as the postulate of neuropsychological reducibility or the postulate of psychophysical causality may influence theory-building, research methodology, as well as diagnostics or even therapeutic interventions (see Fahrenberg, 2013, 404; Fuchs, 2017; Marom, 2020). Explicit metatheoretical frameworks for the subject-matter of these sciences are, for instance, biological naturalism, which regards mental phenomena as properties of the *brain* (Searle, 1992), or enactivism, which holds them as emergent properties of an *organism* in a dynamic-reciprocal interplay with its environment (see Lee, 2023). Krakauer et al. (2017) have claimed that cognitive neuroscientists and psychologists, while guided by philosophical beliefs, implicitly adumbrate the lack of an *explicit metatheoretical or conceptual framework* when they use filler terms. Without such a framework, statements like “The circuit X is *involved* in behavior Y” (ibid., 485) would be a mere restatement of the correlative or causal relation and would not (further) contribute to any explanation. The lack of explicit metatheoretical frameworks coincides with the notion of a neglect of (formal) explanatory theory in psychology (Teigen, 2002; Oberauer and Lewandowsky, 2019; McPhetres et al., 2021; Borgstede, 2022; Wendt and Wolfradt, 2022). We wish to call attention to the influence of different metatheoretical frameworks, as it may be the case, that a single empirical finding can be accounted for by multiple explanatory frameworks. The recourse to parsimony to justify the primacy of framework x over framework y is only logically permitted if it is not made unreflectively based on framework x, otherwise one would be committing the fallacy of a *petitio principii*.

In light of the broad array of philosophical views concerning the problem of consciousness, we do not commit ourself to any particular one. This article investigates the metatheoretical beliefs regarding the relation of physiological and psychological variables, which beliefs inherent to different neuropsychological assessment procedures, such as double dissociation and the concept of reverse experimentation (see Kadlec and van Rooij, 2003).

Our intention is to assert that metatheoretical stances may stimulate improved approaches for addressing specific methodological requirements in neuropsychological research, such as internal and ecological validity. To achieve this objective, we draw upon a phenomenological orientation which can be found in 20th century psychology (Lewin, 1936; Herzog, 1992; but also see Wendt, 2022), philosophy (Gurwitsch, 2010), as well as neuropsychology (Goldstein, 1995; Frisch, 2014a).

## The entanglement of metatheory and methodology in neuropsychological assessment

Our endeavor commences with an analysis of a widely used neuropsychological practice known as double dissociation (DD). The rationale of DD holds that, if a brain lesion *A* leads to the impairment of the psychological function *1* but not of function *2* and a brain lesion *B* leads to the impairment of function *2* but not of function *1*, a relative functional independence of the two brain areas can be assumed (see, e.g., Stone and Davies, 1993, 594). A prototypical example is the dissociation of speech production, impaired in patients with lesions in Broca’s area, and the impairment of speech comprehension, impaired in patients with lesions in Wernicke’s area (see Gazzaniga et al., 2014, 472–474).

One classical presupposition regarding DD is that its validity rests on the metatheoretetical assumption of modularity, eventhough this assumption was subject to extensive critique (Shallice, 1988; Plaut, 1995). It should be emphasized that multiple accounts of modularity exist (cf. Gottschling, 2020). For instance, Shallice (1988, 20) discusses Fodor (1983), whose account of modularity defines a module as a subsystem exhibiting specific characteristics, including domain specificity, innate specification, indecomposability into basic elements, hard-wiredness, computational autonomy, information encapsulation, and a distinctive pattern of development. Fodor argues that modules are “computationally autonomous” in the sense that they operate independently without relying on general-purpose processes from other modules. “Informational encapsulation” refers to the limited access of a module to a specific subset of information within the overall system (Shallice, 1988, p. 20). Shallice critically contends that this conceptualization of modularity may be excessively rigid, considering the subject-matter of neuropsychology. Because of these concerns Shallice adheres to the concept of functional differentiation in regard to subsystems. In accordance with Tulving (1983), Shallice asserts that two subsystems exhibit functional dissimilarity when one system functions independently but potentially less efficiently without the support of the other intact system. In the case of functional dissimilarity, enhancements or suppressions in the operations of one system do not necessarily impact the other system in a similar manner. Accordingly, this functional disparity indicates that the systems operate differently and are governed by distinct principles, at least partially (Tulving, 1983, p. 66). However, it is still common to interpret double dissociation as methodological correlate of the metatheoretical assumption of modulartiy [see for a critique (Shallice, 1988; Plaut, 1995)]. Still, it must be noted, that the concepts of modularity and functional dissimilarity bear relevant similarities. When we speak of ‘modular’ we will adress this wide sense of modularity.

## Reflections on double dissociation

The explanatory paradigm of DD may be subjected to critique, for example, from the phenomenological standpoint of enactivism which has been advanced by Thomas Fuchs. In our view, DD is also consistent with a metatheoretical position Fuchs termed “biological epiphenomenalism”. This approach regards consciousness as a “dispensable varnish” (2017, 227), i.e., views conscious experience as a causally ineffective byproduct of brain processes. DD’s primary focus lies in investigating the influence of brain lesions on behavior or experience, specifically examining how physiological variables affect psychological aspects. However, it does not typically investigate the reverse relationship, where psychological factors affect physiological variables. Fuchs rejects the notion of a dualism between mind and brain that is implied by such perspectives. In his view, psychological variables are not separate from bodily processes. He regards psychological variables as abstractions used to describe properties of an *embodied mind*. For Fuchs, it is the conscious, living organism, which possesses causal power, not the abstraction (2017).

Marom (2015) largely agrees with Fuchses perspective (2015, pp. 49–68). For Marom, psychological and physiological variables are viewed as *categorically, but not necessarily ontologically* distinct (see Fahrenberg, 2013, 2015). It may be argued that DD does not adequately consider this categorical distinction. Furthermore, if DD is approached from a biological epiphenomenalist standpoint, it becomes challenging to reconcile certain empirical findings. Examples of such findings are that subjectively experienced stress is predictive of somatic health outcomes (Tsukerman et al., 2020), that meditation enhances hippocampal connectivity (Lardone et al., 2018), or that psychotherapy improves the linkage between the amygdala and the cognitive control network (Shou et al., 2017). The reason for this explanatory difficulty is that the conceptual framework of biological epiphenomenalism does not accommodate the effects of psychological variables on physiological variables. Enactivism, on the other hand, argues that through *downward causality*, psychological variables, as emergent properties of the *embodied* mind, can influence “simpler” biological variables (Fuchs, 2020). However, the potential for circular causality remains a subject of debate (see, for example, Lee, 2023), and for the purpose of our discussion, we remain true to the metatheoretical perspective by bracketing the decision for one or the other standpoint.

One of has summarized further arguments against DD in a previous article. On the one hand, the aforementioned concept of dissociation of function seems problematic due to a lack of factor independence. Additionally, DD has been subject to criticism for relying on non-experimental *ex post* facto data. Consequently, DD faces limitations in establishing causal relationships between neurobiological and mental phenomena. Moreover, it fails to demonstrate necessary identity between psychological and physiological phenomena on the ontological level due to the existence of an indefinite number of potential neural networks that can implement the same psychological function (Peper, 2018).^1^

Double dissociation but also its critical adversaries, are substantially influenced by their underlying metatheoretical pre-suppositions. This highlights the importance of methodological reflection, as it has the potential to facilitate metatheoretical reconciliation and potential improvement. In the following discussion, we will illustrate how a meta-model for neuropsychological assessment (Peper, 2018), as well as the phenomenological orientation in psychology (Wendt, 2018) and neuropsychology (Goldstein, 1995; Frisch, 2014a,b) might contribute to addressing the limitations of DD and potentially overcome its shortcomings.

## A lens type meta-model for neuropsychological assessment

Within neuropsychological assessment theory, one of us has put forth the *neuro-lens model* (NLM) which is a neuropsychological *generalization* of DD since the latter can be regarded as a special case of the former (cf. Peper, 2018). NLM’s epistemological approach to relate distal and proximal entities draws on the metaphor of the lens (cf. Brunswik, 1952).

The NLM framework poses the following pre-conditions for inferring causal relations between psychological (Ψ) and physiological (Φ) domains incorporates the following three pre-conditions: (a) the ability to experimentally manipulate the psychological and physiological variables of interest, (b) the identification of convergent and discriminatory correlations, which are indicators of validity, and (c) the investigation of both causal directions between psychological and physiological variables, that is, examining the influence of Ψ on Φ (Ψ→Φ) as well as the influence of Φ on Ψ (Φ→Ψ).

According to the logic of this so-called *reverse experimentation approach*, a psychological function of interest could be stimulated to show that a specific biophysical activation depends on that function, and not on another activation. For instance, a visual stimulus could be presented in an fMRI experiment to capture the neural correlates associated with visual perception.^2^ In contrast, neural system manipulation could be utilized to demonstrate the modification of a specific psychological function while leaving others unaffected (Peper, 2018): transcranial magnetic stimulation (rTMS) could be applied, for example, to induce a temporary disturbance in the motor cortex (M1), selectively impacting hand movement in one region and arm movement in another (Peper, 2018).

Methodologically speaking DD can be seen as a specific application of the NLM. The NLM offers methodological advantages, such as its hierarchical multilevel structure, which addresses the issue of factor independence in both mental and physiological variables. In addition to these methodological considerations, the NLM brings about a shift in the metatheoretical assumptions of neuropsychological assessment strategies. According to this, experimental manipulation can be applied to both categorical domains of neural and psychological phenomena. It thus captures the range of possibilities that have been developed within the field of neuropsychological assessment and research and offers a more comprehensive approach to exploring the complexities of brain-mind relationships.

Double dissociation and the NLM both describe methodological procedures, while e.g., epiphenomenalism or the system science/network view are metatheories. Yet, metatheory and methodology are not independent. Because DD (merely formally) can be seen as a special case of the NLM, one could employ DD’s methodology while adhering to a metatheoretical network perspective. However, it is not possible to be a metatheoretical epiphenomenalist and simultaneously employ the NLM as methodological framework.

The generalization by the NLM encompasses methodological and metatheoretical perspectives concerning the context-dependency of psychophysiological variables. This context-dependency, however, may not be adequately addressed within the framework of classical discriminant diagnosis of which DD is an instance. This is particularly the case when this framework is approached from a modularist perspective, which according to Frisch (2014b) often assumes that knowledge acquisition occurs solely within standardized environments. However, methodologically there is no inherent reason why (experimental) research cannot be conducted beyond the confines of the laboratory (Fahrenberg et al., 2007). We therefore see that the metatheory associated with the NLM is preferable to one that does not consider the context and context-dependency of psychological, as well as physiological variables. The NLM emphasizes the context dependency of psychological and physiological attributes with regard to methodology and metatheory.

Concerning the issue of *ecological validity*, Peper follows Brunswik, in stating that “the conditions and materials of assessment should be representative of the environment of the person. Multiple interacting environments, for instance, shared or non-shared contexts of personal life events can be identified. Thus, different types of lens models are needed to improve ecological validity” (Peper, 2018, 272). This assertion seems especially important since the ability of some neuropsychological tests to predict the impairment of patients in their daily living environment appears to be limited (e.g., Peper and Loeffler, 2014; Suchy et al., 2022).

The concept of “ecological validity” has been criticized recently for conceptual vagueness and risk of antagonizing the “real world” and the “neutral lab” (Holleman et al., 2020). Consistent with Peper’s assumptions of *differences in contexts*, phenomenological psychology’s paradigm of *situation analysis* can shed light on the fact that the laboratory is *but one context of experience*, as one of us has argued (Wendt, 2018).^3^ It is crucial to understand, however, that complementary to an understanding of the context of the “physical” environment of an organism, it is also necessary to assume a subjective experienced environment (in the sense of Umwelt the works of theoretical biologist Jakob Johann Von Uexküll, 1921). Among other reasons, because it is possible, that people situated within the same physical environment experience a different *Umwelt* (Gurwitsch, 1976), a *descriptive* approach to the assessment of the situation of an individual may contribute to neuropsychological procedures (cf. Frisch and Métraux, 2021). This perspective thus helps both to avoid the justified criticism by Holleman et al. concerning the antagonization of the “real world” and a supposedly neutral laboratory and to take different types of experienced situations into account (Wendt, 2018, 4). Striving for ecological validity makes it necessary to reflect on metatheoretical stances regarding the contextual nature of the human condition.

## Contextuality and metatheoretical dialogue

Metatheoretical reflections regarding the contextual nature of the human condition can be found in phenomenological psychology, which has a long tradition of emphasizing that human experience is situated (Lewin, 1936; Merleau-Ponty, 1962; Gurwitsch, 2010; Wendt, 2018). The observation that the laboratory, unlike many other contexts of human experience and behavior, is characterized by an elimination of many everyday stimuli does not contradict the observation that contexts outside the laboratory are heterogeneous. In shared work one of us has argued that

[n]atural situations differ from lab situations in multiple ways as they require more complex planning, organizational and monitoring processes. In contrast, lab environments are typically void of distractors that divert the subjects attention from the task. Moreover, the test administrator, who structures the test session and supports the subject throughout the procedure, is not present in real life; thus, a crucial social agent that compensates for deficits and provides extrinsic motivation is absent (Peper and Loeffler, 2014, 233–234).

According to Eling (2015), the phenomenologically oriented physician Kurt Goldstein (1878–1964) spoke of some test situations as being “lebensfremd,” (not true to everyday life) and of others as being “lebenswahr” (true to everyday life). Goldstein, together with the gestalt psychologist Adhémar Gelb (1887–1936), played a central role in the advent of contemporary neuropsychology. Goldstein’s phenomenologically inspired positions can be understood as a metatheoretical or metascientific attempt at structuring the various schools of theory and methods presented here. Accordingly, Goldstein was an early critic of modularity, stressing that psychological functions can only be understood if the *whole organism* is taken into consideration (Gelb and Goldstein, 1920; Rimpau, 2009). This position possesses at least some similarity with enactivism which commonly regards psychological variables as properties of the entire organism (cf. Fuchs, 2017). Frisch (2014a) notes that Goldstein viewed practices contingent on some versions of modularity, as DD according to some authors (Warrington, 1981), as insufficient, because they do not consider that patients can partially regain psychological functions after brain lesion. The possibility of such recovery indicates that extended networks can realize the realization of a psychological function. Furthermore, the realization of a psychological function via a complex system can be disrupted if one damages a *part* of the system.^4^ This does not imply that one can infer a localization of the function within the lesioned part.

Frisch argues, that the loss of a psychological function may be dependent on a situation. For instance, the recall of the same words may be disturbed in the symbolic context (naming) but not in the concrete-emotional context (scolding).^5^ Lastly, Goldstein’s clinical work indicated that brain lesions usually do not affect only a single function. Likewise, it would rarely be the case, that a psychological function is fully absent after lesion, with other psychological functions being completely intact (Frisch, 2014a,b). It seems reasonable to assume that these sophisticated aspects can be better addressed by the generalized NLM than by DD. Goldstein regarded the brain as a *network* (*Netzwerk*) situated within the organism which he again viewed as situated within its life and within a concrete situation (Goldstein, 1927, 1995; Frisch, 2014a,b; Frisch and Métraux, 2021).

The metatheoretical potential of Goldstein’s position lies in the fact that it does not imply that we need to abandon any assumption of local specification at a particular time *t*. Equally, in our opinion, a view of the brain as a dynamic network nested in an organism which is nested in a world is also largely consistent with some versions of modularity. As we have noted, metatheoretical beliefs structure scientific theories; yet, they are not easily falsifiable. Since a lesion rarely leads to a complete loss of psychological function (cf. Frisch, 2014a), one can either argue that the case is not “pure” enough and therefore in favor of modularity or interpret the findings as evidence against modularity.^6^ However, it obviously makes a difference whether the hippocampus or the PFC is affected by a lesion, whether this is due to the modular structure of the brain or to the fact that a part of a circuit has been damaged. Given that modularists must acknowledge the plasticity of the brain, the branch of modularity that seems largely consistent with a system science neuropsychological assessment strategy can be regarded as *dynamic* modularity. Furthermore, Frisch (2014a) emphasizes that Goldstein did not subscribe to equipotentialism, the idea that solely the size of the lesion was of functional importance. Moreover, some authors argue that a network approach to the brain is compatible with versions of modularity (Alexander-Bloch et al., 2010).

We need not settle the question of whether the modularity assumption holds, since, our aim is only to demonstrate that philosophical assumptions have the potential to shape both research and assessment in neuropsychology. In this context, Goldstein’s belief that the brain is a dynamic and adaptable network, and that lesions have a comprehensive impact on the entire organism, which in turn adapts its *Umwelt* to cope with the new situation, aligns with various metatheoretical frameworks in neuropsychology. The adaptation of the organism encompasses not only physical aspects of the environment, but also subjective experiences structured by demand characteristics and affordances (cf. Lewin, 1936; Dings, 2020). By considering the contextual aspects of individuals and patients, both in terms of their distal environment (physical surroundings) and proximal environment (*Umwelt*), generalized lens models might help to effectively examine the relationship between proximal and distal aspects of the subject matter of neuropsychology.

## Conclusion

Our aim was to revisit the metatheoretical or philosophical beliefs that accompany neuropsychological research and assessment. Despite appearing to be in opposition, the classical neuropsychological approach of DD can be understood either as assuming localizationism and a modular view of the brain, or as a specific case of lens-type modeling approaches (NLM) to neuropsychological assessment. The latter interpretation more readily aligns DD with a comprehensive systemic view of the human brain as a network that, as part of the whole organism, mediates the situatedness of human beings in the world. These perspectives closely intersect with the empirical and theoretical work of early neuropsychologist Kurt Goldstein, who emphasized the situatedness of the organism within its *Umwelt* (subjectively experienced environment). Thus, both modularity and the system science approach sketched here, converge in Goldstein’s claim that the brain is a dynamic and adaptable network, and that lesions impact the entire organism, which then adapts its *Umwelt* to cope with the new overall situation. This perspective not only enables the structuring of ecological validation processes through a metatheoretical foundation, but also aligns with the idea from phenomenological psychology, that the laboratory is only one of many situations. Lens-type models may provide a methodological framework to better adapt neuropsychological assessment strategies, that accommodate a minimal consensus among the different metatheories of neuropsychology. The analysis therefore shows that metatheory in neuropsychology is not in opposition to therapeutic practice and research. All three levels are in epistemic continuity and can complement each other in a substantial manner.

## Data availability statement

The original contributions presented in this study are included in the article/supplementary material, further inquiries can be directed to the corresponding authors.

## Author contributions

JR developed the initial idea and wrote the first manuscript. AW and MP made substantial contributions to the text. JR, AW, and MP jointly finalized the manuscript. All authors contributed to the article and approved the submitted version.

## Conflict of interest

The authors declare that the research was conducted in the absence of any commercial or financial relationships that could be construed as a potential conflict of interest.

## Publisher’s note

All claims expressed in this article are solely those of the authors and do not necessarily represent those of their affiliated organizations, or those of the publisher, the editors and the reviewers. Any product that may be evaluated in this article, or claim that may be made by its manufacturer, is not guaranteed or endorsed by the publisher.

## Footnotes

1 A note for the philosophically inclined: The argument of *ex post* facto data is especially relevant to non-identity theorists. The argument concering necessary relation is especially relevant to identity theorists. Pepers critique thus remains forceful from different metatheoretical standpoints.

2 It should be noted, however, that identifying the substrate, i.e., the correlating brain state of a psychological function, is a difficult undertaking. Every state of consciousness is accompanied by its neural enabling conditions, its neural substrate, and its neural consequences. de Graaf et al. (2012) argue that only enabling conditions and consequences can be separated from each other, while the assumed substrate of mental function always remains intertwined with one of the two and thus eludes identification in empirical analysis.

3 The acknowledgment of the laboratory as a meaningful situation, governed at least partially by, among other experiential factors, social rules, and individual expectations, may create an opportunity to analyze, for instance, the Milgram Experiment as an investigation into the authoritative role of science in Western societies (see Haslam et al., 2014). Overlooking the fact that the act of entering a laboratory stimulates a distinct experience may result not only in an overestimation of the generalizability of experimental results but eventually also leads to impaired interpretations of empirical findings.

4 It has been argued that this was demonstrated by von Monakow (cf. Frisch, 2014a).

5 According to Frisch (2014a), this was demonstrated by Hougling Jackson.

6 Van Orden et al. claim that the first interpretation leads to the iterative introduction of new modules, as there are no criteria for the acceptance or rejection of modules (cf. 2001).
